# Supplementary material for: Identification and Quantitation of Novel ABI3 Isoforms Relative to Alzheimer’s Disease Genetics and Neuropathology
Source: Genes (Basel). 2022 Sep 8;13(9):1607. doi: 10.3390/genes13091607 (PMC9498898; doi:10.3390/genes13091607)
Supplement: Supplementary file 1 [file genes-13-01607-s001.zip › genes-1878555-supplementary.pdf]

Table S1. Subject metadata for samples used in this study.

Sample Number	Age at death (years)	Sex	Braak Score	NIARI Score	PMI (hours)
1	105	Male	III	No likelihood	3.0
2	92	Female	II	Low likelihood	2.3
3	65	Female	O	No likelihood	3.0
4	80	Male	I	No likelihood	3.5
5	72	Male	I	No likelihood	0.0
6	66	Female	I	No likelihood	4.5
7	85	Male	III	Low likelihood	2.0
8	84	Female	III	Intermediate likelih	1.5
9	79	Male	II	Low likelihood	2.3
10	80	Male	IV	No likelihood	3.0
11	75	Female	I	No likelihood	3.5
12	86	Female	II	Low likelihood	2.3
13	95	Male	I	No likelihood	1.8
14	82	Female	II	No likelihood	3.0
15	77	Male	I	Low likelihood	3.5
16	87	Male	II	Low likelihood	2.0
17	72	Female	I	No likelihood	3.8
18	91	Female	I	No likelihood	4.0
19	86	Female	I	Low likelihood	3.8
20	81	Male	II	Low likelihood	2.0
21	82	Male	I	Low likelihood	2.1
22	74	Male	I	No likelihood	4.0
23	75	Male	V	High likelihood	4.0
24	89	Female	IV	Intermediate likelih	1.8
25	84	Female	IV	Intermediate likelih	2.5
26	79	Male	II	Intermediate likelih	1.8
27	87	Female	IV	Intermediate likelih	2.4
28	91	Female	II	Low likelihood	1.8
29	79	Female	VI	High likelihood	3.0
30	84	Female	VI	High likelihood	2.8
31	80	Female	VI	High likelihood	4.5
32	83	Male	VI	High likelihood	4.0
33	88	Male	VI	High likelihood	2.8
34	83	Male	VI	High likelihood	4.0
35	86	Female	VI	High likelihood	4.3
36	77	Male	VI	High likelihood	3.5
37	84	Female	VI	High likelihood	3.3
38	73	Male	VI	High likelihood	2.8
39	81	Male	VI	High likelihood	3.8
40	90	Female	VI	High likelihood	2.8
41	78	Male	VI	High likelihood	3.8
42	80	Female	VI	High likelihood	2.8
43	69	Male	VI	High likelihood	4.0
44	95	Male	VI	High likelihood	4.0
45	86	Female	VI	High likelihood	3.3

46	84	Female	VI	High likelihood	5.0
47	90	Female	VI	High likelihood	2.8
48	82	Female	VI	High likelihood	2.5
49	86	Female	VI	High likelihood	3.3
50	90	Male	VI	High likelihood	3.3
51	78	Male	VI	High likelihood	3.5
52	68	Female	VI	High likelihood	3.3
53	83	Female	VI	High likelihood	3.5

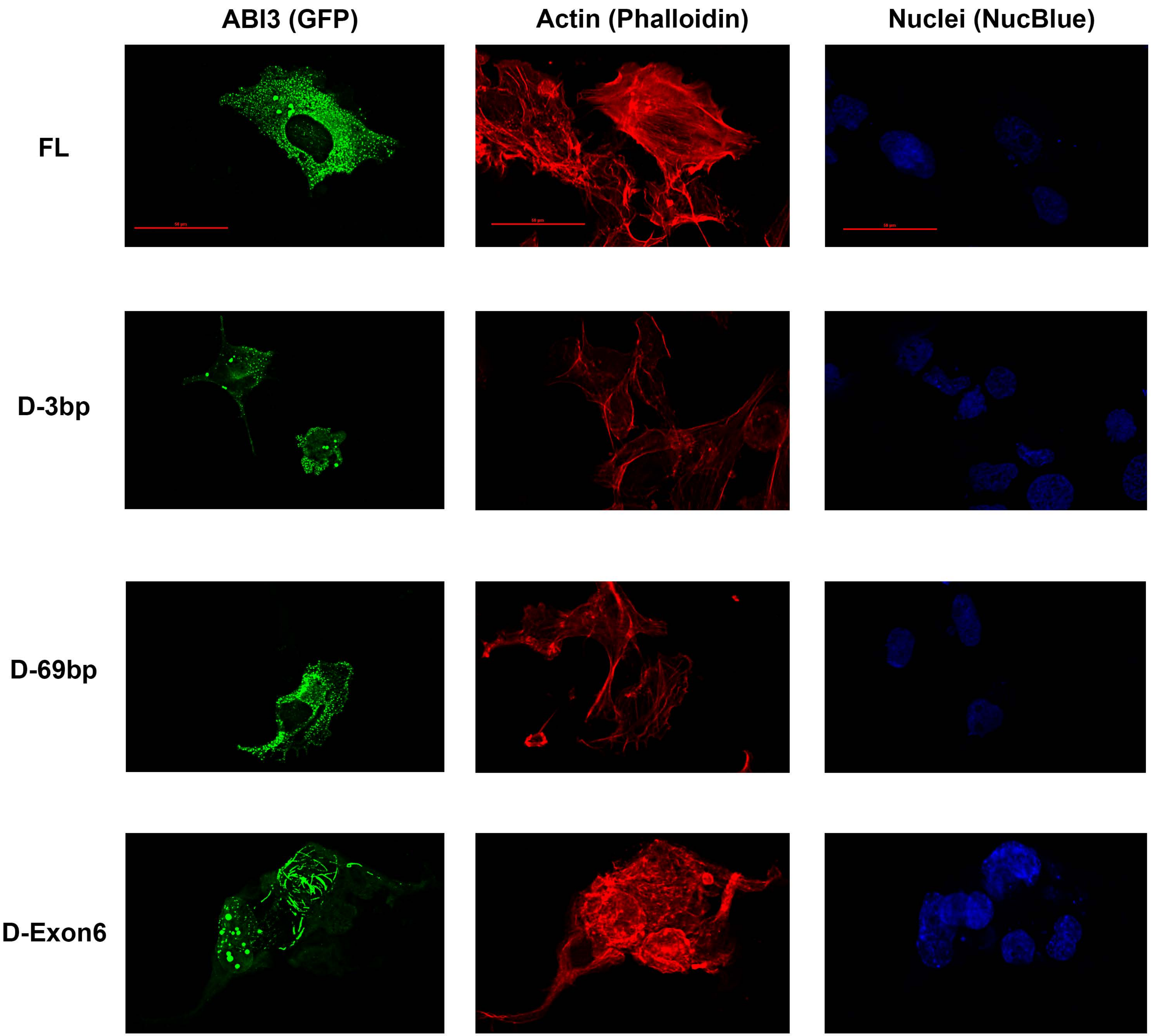

Figure S1. Color-separated ABI3 localization.
